# Application of Micro-Western Array for Identifying Different Serum Protein Expression Profile among Healthy Control, Alzheimer’s Disease Patients and Patients’ Adult Children

**DOI:** 10.3390/brainsci12091134

**Published:** 2022-08-26

**Authors:** Chieh Huo, Ming-Hui Chen, Tzyh-Chyuan Hour, Ling-Chun Huang, Yi-On Fong, Ying-Yu Kuo, Yuan-Han Yang, Chih-Pin Chuu

**Affiliations:** 1 Institute of Cellular and System Medicine, National Health Research Institutes, Miaoli 35053, Taiwan; 2 Neuroscience Research Center, Kaohsiung Medical University, Kaohsiung City 80708, Taiwan; 3 Department of Biochemistry, School of Medicine, Kaohsiung Medical University, Kaohsiung City 80708, Taiwan; 4 Department of Neurology, Kaohsiung Municipal Ta-Tung Hospital, Kaohsiung Medical University, Kaohsiung City 80145, Taiwan; 5 Department of Neurology, Kaohsiung Medical University Hospital, Kaohsiung City 80756, Taiwan; 6 School of Post-Baccalaureate Medicine, Kaohsiung Medical University, Kaohsiung City 80708, Taiwan; 7 Department of Life Sciences, National Central University, Taoyuan City 32031, Taiwan; 8 PhD Program for Aging, Graduate Institute of Basic Medical Science, China Medical University, Taichung City 40402, Taiwan; 9 Biotechnology Center, National Chung Hsing University, Taichung City 40227, Taiwan

**Keywords:** Alzheimer’s disease, Micro-Western Array, ABCA1, ABCG1, ApoD, ApoE, COX2, SREBP2, YAP

## Abstract

(1) Background: Alzheimer’s disease (AD) is the most common form of dementia. Increased levels of inflammatory proteins have been observed in brain and plasma samples of AD patients; however, it is not clear if other serum proteins correlate to the development or disease progression of AD. (2) Methods: Micro-Western Array (MWA) is a high-throughput antibody-based proteomics system which allows detection of the expression levels of 24–96 different proteins within 6–30 samples simultaneously. We applied MWA to explore potential serum protein biomarkers correlated to the development and progression of AD by examining the difference in serum protein profile of 31 healthy control (HC), 30 patients with AD and 30 patients’ adult children (ACS). (3) Results: Compared to HC, AD and ACS express similar pattern of serum proteins, including higher protein levels of ABCA1, ABCG1, SREBP1 and LXRβ but lower protein levels of ApoD, ApoE, ApoH, c_Myc, COX2 and Hippo-YAP signaling proteins. AD patients had higher serum levels of ABCG1, ApoD, ApoH, COX2, LXRα and YAP, but lower levels of ABCA1, ApoE, c_Myc, LATS1, MST1, MST2, Nanog, NFκB_p50, PPARγ and SREBP2, as compared to ACS. Pearson’s correlation analysis revealed that the protein expression level of ApoE, c_Myc, LATS1, MST2, NFκB p50, PPARγ and SREBP1 was negatively correlated to age, while that of ApoE, c_Myc, LATS1, MST1, MST2, Nanog, NFκB p50 and PPARγ was positively correlated to age. (4) Conclusions: We identified a group of serum proteins which may correlate to disease progression of AD and can be potential diagnostic serum protein biomarkers.

## 1. Introduction

Dementia is currently the 7th leading cause of mortality globally and Alzheimer’s disease (AD) is the most common form of dementia. Approximately 47 million people have been diagnosed with AD in the world. AD is a chronic brain disorder characterized by progressive decline of intelligence and loss pf memory and neuron. These changes are caused by extracellular deposition of amyloid-β (Aβ) and intracellular accumulation of hyperphosphorylated tau protein in prefrontal cortex and hippocampus [1]. Deposition of amyloid-β peptide drives cerebral neuroinflammation by activating microglia. Activation of inflammatory pathways is one of the main causes for AD, as people taking nonsteroidal anti-inflammatory drug (NSAID) show a reduced risk for developing AD [2]. Microglia is the resident macrophages of the brain, while astrocytes are the most abundant cells supporting neurons in the brain. Activated microglia and astrocytes secrete lots of pro-inflammatory cytokines, chemokines, reactive oxygen species (ROS), nitric oxide (NO) prostaglandins, etc. [2]. Astrocytes can be activated in the presence of Aβ. Aβ-induced astrocytes overexpress several inflammatory related factors, including IL-1β, TNF-α, inducible NO synthase (iNOS), and NO [2]. NLRP3 (NOD-, LRR- and pyrin domain-containing protein 3), predominantly expressed in macrophage, acts as a sensor molecule, and forms the NLRP3 inflammasome together with the adaptor protein ASC and pro-caspase-1 [3,4]. NLRP3 is critical for the innate immune system and activation of NLRP3 inflammasome leads to caspase 1-dependent release of the pro-inflammatory cytokines IL-1β and IL-18, as well as to gasdermin D-mediated pyroptotic cell death [3]. Expression of NLRP3 is elevated in brains of AD patients and activation of NLRP3 inflammasome enhances Aβ aggregation by reducing Aβ phagocytosis [5]. Inhibition of NLRP3 inflammasome function reduced tau hyperphosphorylation and aggregation [6]. High plasma level of α1-antichymotrypsin (ACT), IL-1β, IL-2, IL-4, IL-6, IL-10, TNF-α, G-CSF, TGF-β1, interferon γ (IFN-γ) and C-reactive protein (CRP) are associated with increased risk of AD [7,8,9]. However, it is not clear if other serum proteins correlated to the development or disease progression of AD.

**Figure 1:**
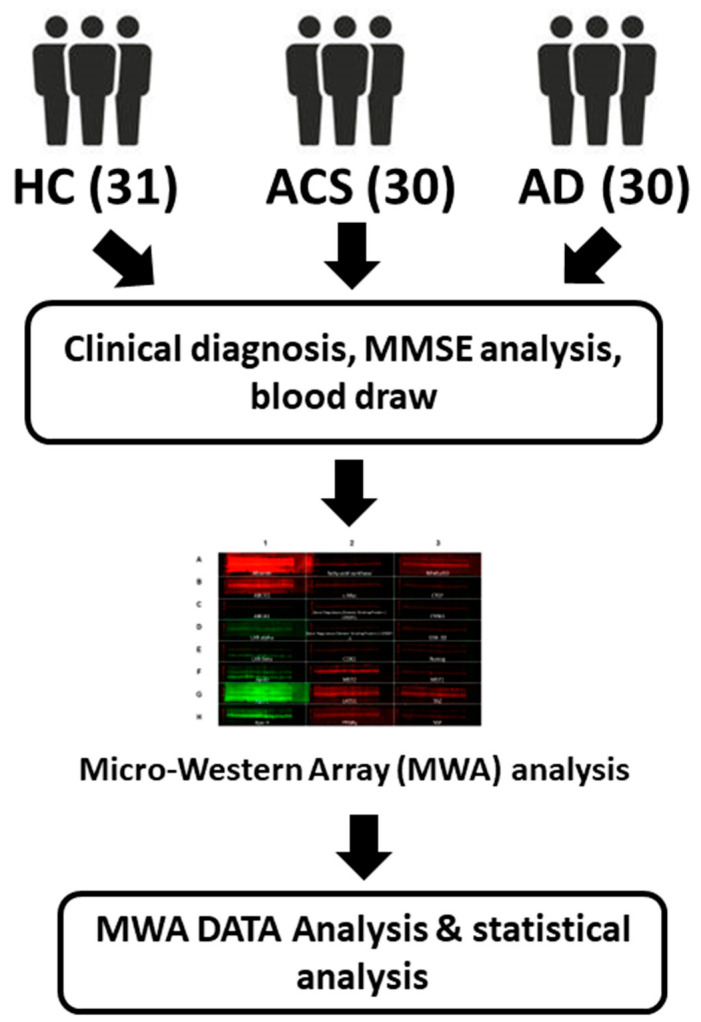
Experimental design. 31 healthy control (HC), 30 patients with AD (AD) and 30 adult children of AD patient study (ACS) were recruited in this study and were being examined with clinical doctors for health status, MMSE, ApoE ε4 expression, and blood draw. The serum samples were then being processed and analyzed with Micro-Western Array (MWA). The protein expression levels was then analyzed for correlation with age or MMSE.

We have previously established a high-throughput antibody-based proteomics system named Micro-Western Array (MWA) [10]. MWA is a modified reverse phase array composing of a GeSim Nanoplotter arrayer, a GE multiphor and a Licor Odyssey infra-red scanner. MWA platform can detect protein expression or phosphorylation status of 24–96 different antibodies in 6–30 samples simultaneously. This novel proteomics technology is a useful systems biology tool to study signaling transduction network and protein profile. We hypothesized that the profile of serum protein be different in healthy control (HC), patients with AD (AD), and adult children of AD patient study (ACS). These proteins can be a useful potential diagnostic biomarker for the development of AD. We therefore applied the MWA to explore the difference in serum protein profile of proteins involved in LXR signaling, apolipoproteins, Hippo-YAP signaling, and inflammation-related proteins in 31 HC participants, 30 AD participants and 30 ACS participants. The experimental procedure was shown in Figure 1.

## 2. Results

### 2.1. Micro-Western Array Reveals the Difference in Serum Proteins Profile between Healthy Control versus ACS and AD

**Figure 2:**
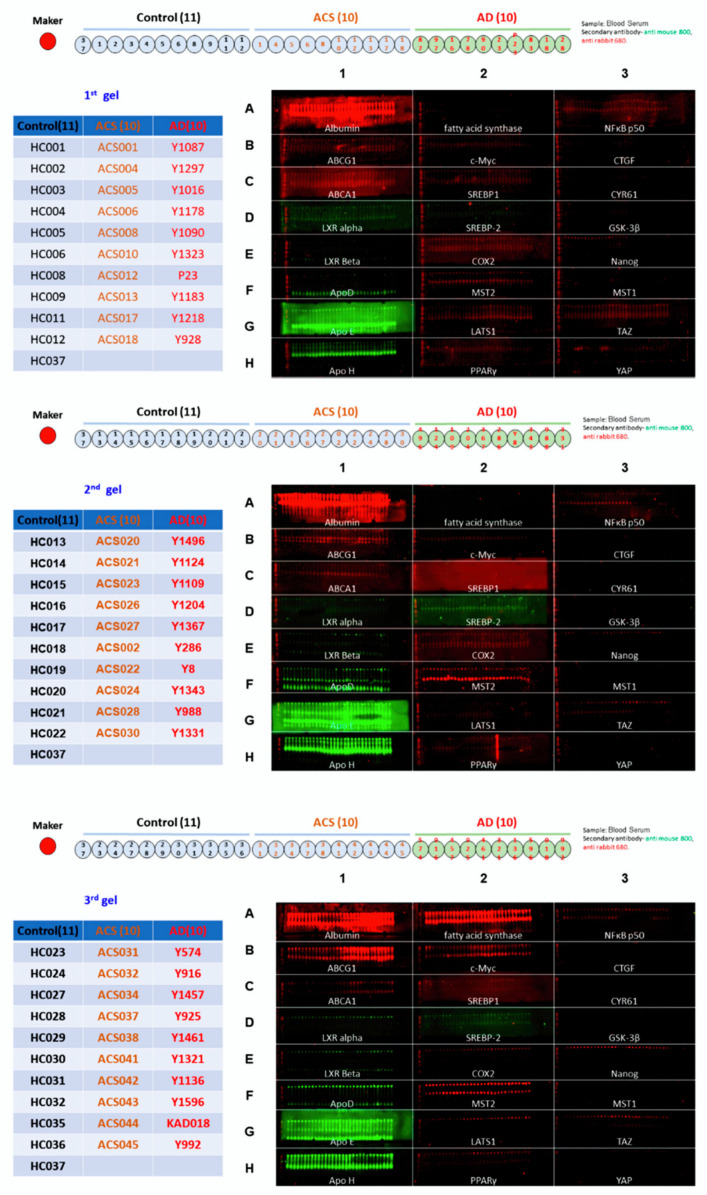
Images of Micro-Western Array analysis for analysis of serum protein expression profile in HC, ACS, and AD participants. The 31 health control samples, 30 ACS samples and 30 AD samples were separated into three MWA gel loading for analysis as gel 1, gel 2 and gel 3. The image showed the loading location of 11 healthy control, 10 ACS and 10 AD in each gel. Sample HC37 was loadined in each gel as standard for comparison between different blots. Red color and green color represented the two different laser in Licor scanner (680 nm and 780 nm wavelength, respectively) used to detect anti-rabbit and anti-mouse antibodies. The albumin was used as loading control. The samples were spotted into 24 wells and were labeled with row 1 to row 3 and column A to column H. In each well, the identical 30 samples were spotted by GeSim Nanoplotter and each well was loaded with one specific antibody as shown in the figure.

**Figure 3:**
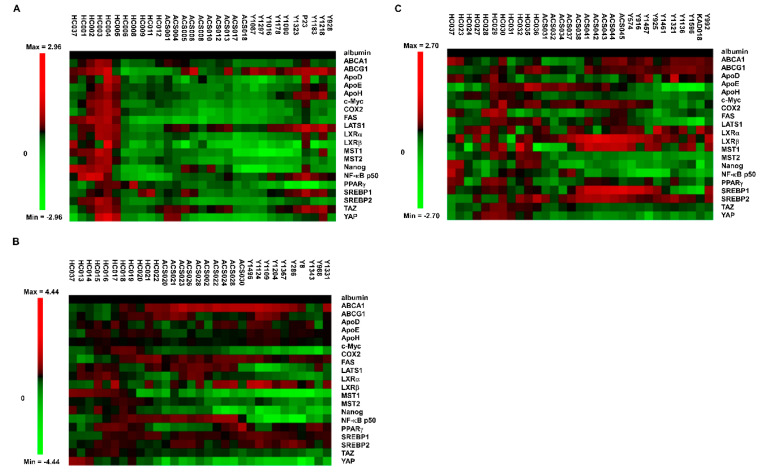
Micro-Western Array analysis of serum protein expression profile in HC, ACS and AD participants. The 31 health control samples, 30 ACS samples and 30 AD samples were separated into three MWA gel loading for analysis as gel 1 (**A**), gel 2 (**B**) and gel 3 (**C**). The average expression level of a specific protein in HC group was used as standard for that protein and was set to be one for relative protein abundance. Expression of proteins in all participants was normalized to the standard level and was converted to log_2_ value for heatmap demonstration. Red color indicated an increase while green color represented an decrease as compared to the standard. The albumin was used as loading control.

**Figure 4:**
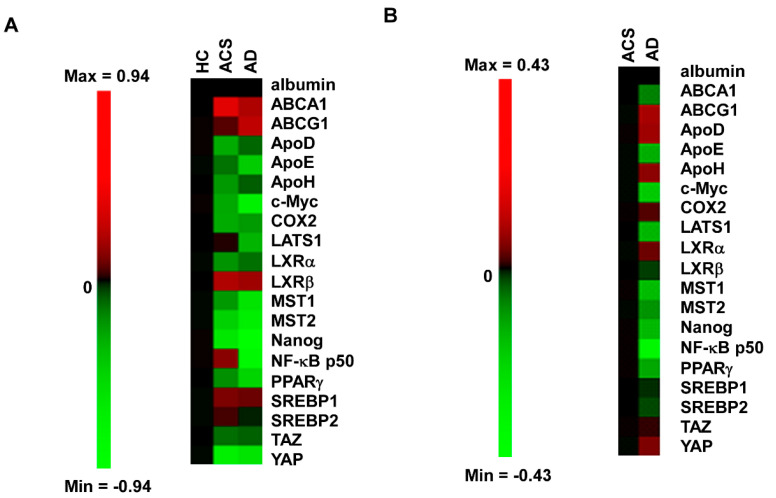
Expression profile of serum proteins in HC, ACS and AD participants as determined by Micro-Western Array. (**A**) The profile of serum protein profile in 31 health control samples, 30 ACS samples and 30 AD samples was demonstrated by heatmap. The average of relative expression level of the serum proteins in all 31 HC, 30 ACS and 30 AD. The heatmap was shown as log_2_ value. (**B**) The profile of serum protein profile in 30 ACS samples and 30 AD samples was demonstrated by heatmap. The heatmap was shown as log_2_ value. Red color indicated an increase while green color represented an decrease as compared to the standard.

We recruited 31 HC (healthy control), 30 AD (patients with Alzheimer’s disease) and 30 ACS (adult children of AD patient study) in this study. The demographic and clinical characteristics of these studied participants were listed in Table 1. The ACS population had the youngest age and longest education years. AD and ACS population had a similar percentage of APOE ε4 positive rate, which was approximately three times higher than that of HC population. The majority of participants in AD and ACS groups were female while 2/3 of the participants in HC were female. The ACS group had the highest MMSE score. We applied Micro-Western Array (MWA) with 24 antibodies to examine the difference in profile of serum proteins in these 31 HC, 30 AD and 30 ACS participants (Figure 2). We selected the antibodies used in MWA focusing on apolipoprotein (ApoD, ApoE, ApoH), liver x receptor (LXR) signaling related proteins (LXRα, LXRβ, ABCA1, ABCG1, SREBP1, SREBP2), Hippo-YAP signaling related proteins (YAP, TAZ, MST1, MST2, LATS1, CTGF, CYR61), other metabolism-related proteins (c_Myc, fatty acid synthase, PPARγ), inflammation-related proteins (COX-2, NFκB_p50 ) and Nanog (Figure 3). Compared to the HC, the expression profiles of serum proteins in ACS were more similar to those in AD (Figure 4A). The ACS and AD expressed higher serum levels of ABCA1, ABCG1, LXRβ and SREBP1 but lower serum levels of ApoD, ApoE, ApoH, c_Myc, COX2, LXRα, MST1, MST2, Nanog, PPARγ, TAZ and YAP. These observations suggested that these proteins may correlated to the development of Alzheimer’s disease. 

### 2.2. Profiles of Serum Proteins in ACS and AD Are Different as Determined by MWA

Considering the fact that ACS have not yet developed AD, we suspected that there was a difference in serum protein expression profile between ACS and AD. Indeed, AD patients had higher serum levels of ABCG1, ApoD, ApoH, COX2, LXRα and YAP, but lower levels of ABCA1, ApoE, c_Myc, LATS1, MST1, MST2, Nanog, NFκB_p50, PPARγ and SREBP2 (Figure 4B).

### 2.3. Correlation of Serum Proteins with MMSE or Age

**Figure 5:**
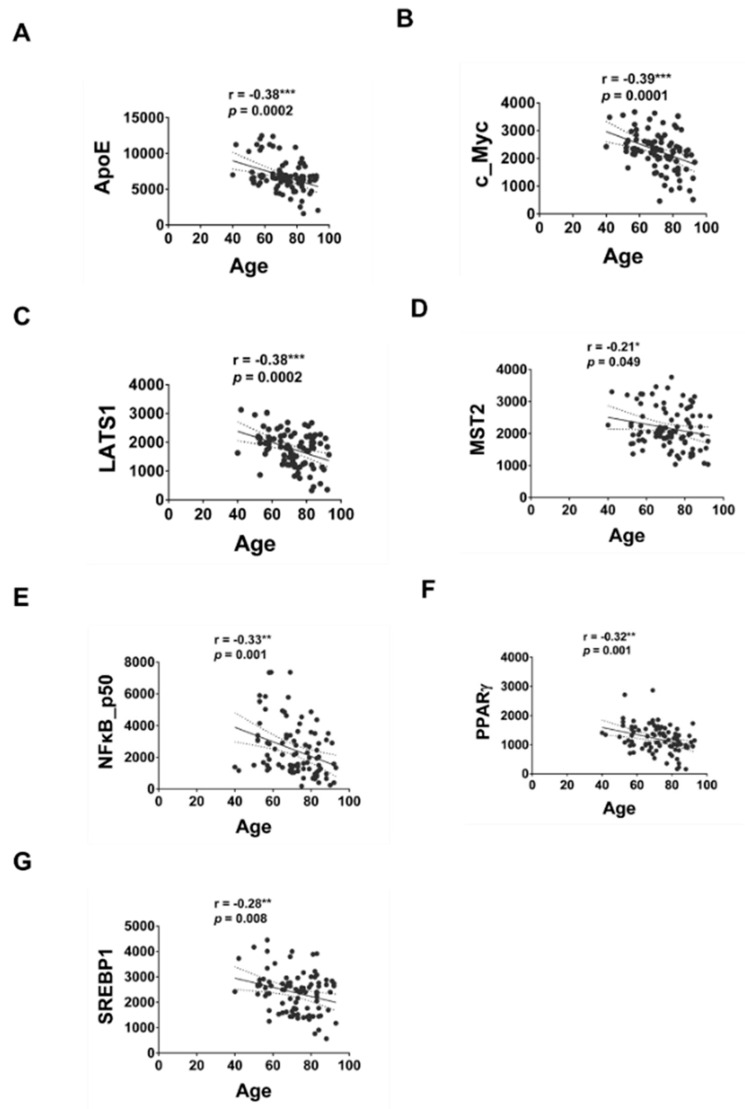
The correlation of expression levels of serum protein and age. Pearson’s correlation test was performed to analyze the correlation between the relative expression level of serum proteins with age for all groups of participants. A two-tailed *p*-value of <0.05 was considered to indicate a statistical significance. Proteins with statistically significant correlation with age was shown for ApoE (**A**), c_Myc (**B**), LATS1 (**C**), MST2 (**D**), NFκB_p50 (**E**), PPARγ (**F**) and SREBP1 (**G**). Asterisks *, **, *** represented a statistically significant difference of *p* < 0.05, *p* < 0.01, and *p* < 0.005, respectively.

**Figure 6:**
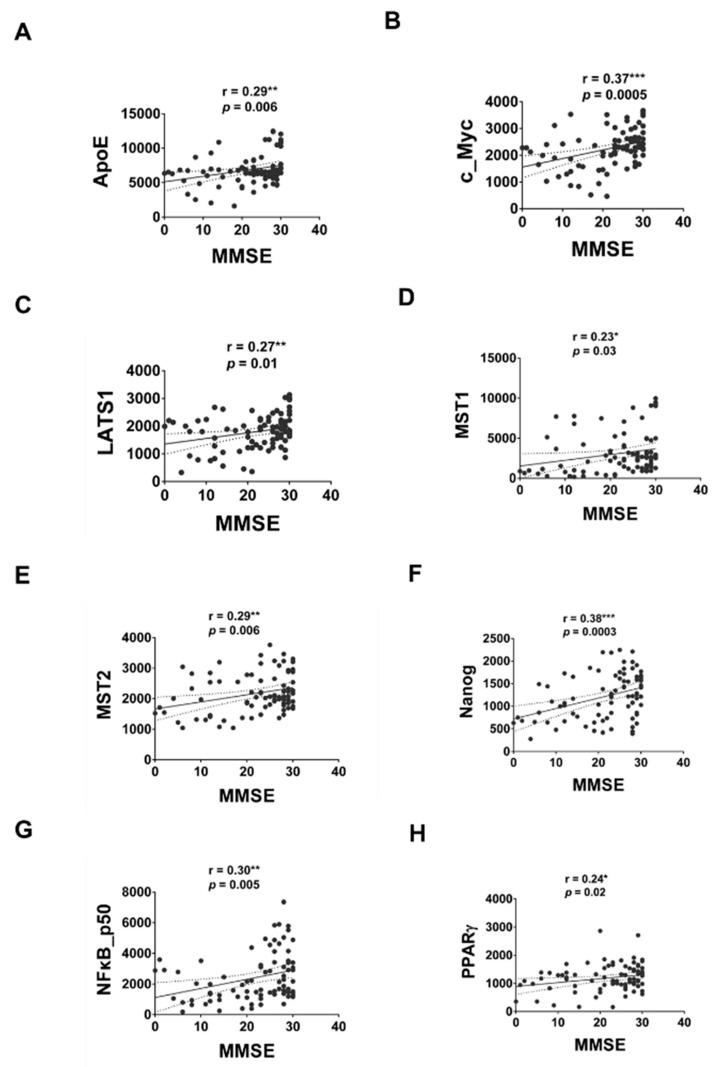
The correlation of expression levels of serum protein and MMSE. The relative expression level of serum proteins and MMSE of the 31 HC, 30 AD and 30 ACS were pooled together for Pearson’s correlation test. A two-tailed *p*-value of < 0.05 was considered to indicate a statistical significance. Proteins with statistically significant correlation with MMSE was shown for ApoE (**A**), c_Myc (**B**), LATS1 (**C**), MST1 (**D**), MST2 (**E**), Nanog (**F**), NFκB_p50 (**G**) and PPARγ (**H**). Asterisks *, **, *** represented a statistically significant difference of *p* < 0.05, *p* < 0.01, and *p* < 0.005, respectively.

We next performed Pearson’s correlation analysis to determine if expression of any of these serum proteins correlate to age or MMSE. The correlation between the expression levels of serum proteins being examined by MWA with age or MMSE was shown in Table 2. The expression of ApoE, c_Myc, LATS1, MST2, NFκB p50, PPARγ and SREBP1 was negatively correlated to age (Figure 5). The expression of ApoE, c_Myc, LATS1, MST1, MST2, Nanog, NFκB p50 and PPARγ was positively correlated to age (Figure 6).

## 3. Discussion

Alzheimer’s disease (AD) is caused mainly by extracellular deposition of β-amyloid (Aβ) plaques and neurofibrillary tangles containing hyperphosphorylated tau protein in the prefrontal cortex and hippocampus, regions essential for memory and learning [1,11]. In this study, we applied Micro-Western Array to analyze the relative expression level of serum proteins in 31 HC, 30 AD and 30 ACS. We included antibodies targeting apolipoprotein (ApoD, ApoE, ApoH), LXR signaling related proteins (LXRα, LXRβ, ABCA1, ABCG1, SREBP1, SREBP2), Hippo-YAP signaling related proteins (YAP, TAZ, MST1, MST2, LATS1, CTGF, CYR61), other metabolism-related proteins (c_Myc, fatty acid synthase, PPARγ), inflammation-related proteins (COX-2, NFκB_p50 ) and Nanog in MWA. We observed that ACS and AD participants shared similar expression pattern of serum proteins as compared to HC. The ACS and AD expressed higher serum levels of ABCA1, ABCG1, LXRβ and SREBP1 but lower serum levels of ApoD, ApoE, ApoH, c_Myc, COX2, LXRα, MST1, MST2, Nanog, PPARγ, TAZ and YAP as compared to HC. However, there was also a difference between AD and ACS. AD patients had higher serum levels of ABCG1, ApoD, ApoH, COX2, LXRα and YAP, but lower levels of ABCA1, ApoE, c_Myc, LATS1, MST1, MST2, Nanog, NFκB_p50, PPARγ and SREBP2 as compared to ACD. Our finding that AD participants and ACS participants share a similar serum protein pattern may reflect the fact that the ACS population has a higher risk to develop AD as compared to HC participants. Some of the ACS participants have reported cognitive decline symptoms, however, their status were still within normal range after evaluation. It is possible that some of these ACS participants may develop AD in future. The observation that the serum protein profile of ACS participants was different from AD participants in certain proteins suggested that these proteins might play important roles in the development of AD, as the ACS population has not developed AD yet. Indeed, previous studies revealed that APOE, ABCA1, ABCG1, ApoD, COX2, SREBP2 and YAP were involved in the disease progression of AD.

APOE ε4 allele e4 is the most well-known risk factor in AD as identified by genome-wide association studies (GWAS) [12]. Activation of APOE triggers an inflammatory cascade that weakens the blood brain barrier (BBB) via an inflammatory molecule known as cyclophilin A (CypA) [13]. Elevation of CypA activates a pro-inflammatory pathway which leads to the BBB breakdown [13]. ApoE is the target gene of LXR. LXRα and LXRβ are members of a nuclear receptor superfamily and use oxysterol as ligands. LXRs maintain the homeostasis of cholesterol efflux and fatty acid synthase. LXRs also provides neuroprotective effects and lowers neuroinflammation. Homeostasis of cholesterol synthesis and efflux is important for maintaining brain functioning [14]. The brain contains a huge amount of cholesterol (~25%) and dysregulation of cholesterol metabolism has been reported in AD [15]. Activation of LXR and retinoid X receptor (RXR) induces LXR target gene ABCA1, leading to Aβ clearance. APOE directly interacts with soluble and fibrillar Aβ [11]. ApoE is involved in cerebral vascular function maintenance, neuro-inflammation, lipid and glucose metabolism, as well as the disease progression of AD [15]. ApoE impairs Aβ clearance and increases amyloid formation [15]. APOE4 promotes seeding and aggregation of oligomers and fibrils Aβ and hinders the clearance of Aβ [11,16]. In AD patients, astrocytes and microglia react with Aβ plaques, cerebral amyloid angiopathy-laden arteries and capillaries, as well as neurofibrillary tangles, and therefore activate transcription of APOE in microglia, down-regulation in astrocytes, and dysregulation of lipid metabolism [11,17,18]. The function of ApoE in the central nervous system (CNS) differs from that of ApoE in peripheral blood, and individuals expressing APOE4 express lower levels of APOE in the CNS as compared to individuals expressing APOE3 [19].

On the other hand, activation of ABCA1, another LXR target gene, leads to amyloid β clearance. ABCA1 and ABCG1 belong to the ATP-binding cassette protein family and are target genes of LXRs. In plasma and CNS, ABCA1 transports cellular cholesterol and phospholipids onto high density lipoproteins (HDL) [20]. ABCA1 is the most abundant CNS apolipoprotein [21]. ABCA1 is required for normal ApoE levels in CNS, lipidation of astrocyte-secreted ApoE, and is involved in cholesterol homeostasis of brain by exporting cholesterol through the blood-brain barrier [21,22]. ABCA1 knockout mice showed decrease of neurite length, reduction of neurite segments, shorter branches of neurites, impaired learning and memory retention and increase of β-amyloid deposition in AD mouse model [20,23]. Deficiency of ABCA1 promotes AD-like phenotype in mice expressing human ApoE4 [24]. Overexpression of ABCA1 protein reduces deposition of Aβ in AD mouse model [25]. Activation of ABCA1 with LXR or RXR agonists increase the lipidation of ApoE4, reverses the ApoE4-driven accumulation of Aβ and tau hyperphosphorylation in hippocampal neurons, as well as improving the impairment of neuron synapses and cognition in AD mouse models [26,27,28]. Loss-of-function mutation in ABCA1 is found to be involved in familial HDL-deficiency, low ApoE plasma levels and a higher risk for AD and cerebrovascular disease [29]. AD patients showed dysfunction of cholesterol efflux capacity of cerebrospinal fluid mediated by ABCA1 and ABCG1 [30]. ABCG1 suppresses activity of γ-secretase and production of β-amyloid [31].

Apolipoprotein D (ApoD) is a lipocalin which can protect tissues against oxidative stress, and elevation of ApoD expression is observed in brain tissue of AD patients [32]. ApoD has been found to be strongly associated with deposits of amyloid in vessels but not with amyloid plaques in human brain tissue. Level of ApoD correlates with age and is higher in men than women, in people older than 50 [33]. Loss of ApoD increases the accumulation of hippocampal amyloid plaque, while overexpression of neuronal ApoD reduces hippocampal amyloid plaque [34].

Expression of cyclooxygenase-2 (COX-2), an enzyme involved in inflammation and neuronal activities, is increased in frontal cortex of AD patients [17]. Peroxidase activity of COX-2 cross-links Aβ and generates Aβ-COX-2 oligomers [35]. Neurons in the brains of AD patients and AD mice show reduction of sphingosine kinase1 (SphK1), leading to defective microglial phagocytosis, decreased secretion of specialized proresolving mediators (SPMs) and reduction of acetyl-CoA-dependent cytoplasmic acetyltransferase activity towards COX2 [36].

Sterol regulatory element-binding proteins (SREBPs) are transcription factors regulating the synthesis of cholesterol and fatty acid. Healthy ApoE4 allele carriers with homozygous SREBP1a ΔG allele have lower risk for AD [37]. Overexpression of SREBP2 in APP/PS1 mice caused early mitochondrial cholesterol loading and mitochondrial glutathione (mGSH) depletion, β-secretase activation and Aβ accumulation, correlating with oxidative damage and neuroinflammation [38]. These mice also exhibited loss of synaptophysin and death of neuron, resulting in impairment of early recognition memory and spatial memory. Tau pathology was also observed in these mice [38].

In hippocampal astrocytes of aging mice and AD model mice, expression of Yes-associated Protein (YAP) decreased and Hippo-Yap signaling was inactivated [39]. YAP prevents premature senescence of astrocytes and cognitive decline of through regulating CDK6 in AD murine model [39]. In addition, neuronal necrosis induced by YAP deprivation occurs before the aggregation of Aβ and regulates AD disease progression in AD murine model [40].

Pearson’s correlation test was performed to analyze the correlation between the relative expression level of serum proteins with either age or MMSE for all groups of participants. We observed that the serum protein level of ApoE, c_Myc, LATS1, MST2, NFκB p50, PPARγ and SREBP1 was negatively correlated to age. On the other hand, the serum protein level of ApoE, c_Myc, LATS1, MST1, MST2, Nanog, NFκB p50 and PPARγ was positively correlated to MMSE scores. PPARγ expression and activity was decreased during aging in rodents [41] and treatment with PPARγ agonist rosiglitazone improved cognition and memory in patients with mild to moderate AD [42,43]. Decreased expression of c_Myc protein and c_Myc downstream signaling increased longevity and health in murine model [44]. NF-κB signaling has been reported to increase with age, and elevation of NF-κB signaling is related to the development and progression of several aging-associated diseases [45]. A previous study of 55 AD and 30 mild cognitive impairments (MCI) reported that no significant correlation was observed between serum ApoE level and MMSE [46]. Although no studies have been done on the correlation between serum levels of c_Myc, LATS1, MST2, NFκB_p50, PPARγ or SREBP1 with MMSE, our study suggested a correlation of the expression level of these proteins in serum with MMSE and age. It will be interesting to further investigate the roles of LATS1, MST2, Nanog and SREBP1 in aging and the development of dementia.

Although the expression levels and function of APOE, ABCA1, ABCG1, ApoD, COX2, SREBP2 and YAP proteins in serum of peripheral blood are different from that in brain tissues, these candidate serum protein biomarkers can be easily examined in blood samples by ELISA or Western blot. Due to the limitation of serum samples being collected at different time points for years, the current MWA data is not able to distinguish the different severity status of AD disease. However, we are currently planning a long-term study to monitor and to collect serum samples of AD participants with different severity of AD diseases, as well as the ACS participants, along with a long-term monitoring and blood draw of other participants with mild cognitive impairment (MCI). We believe that with these samples, the MWA platform can identify the serum proteins correlated to severity of AD or serum proteins that can distinguish the stable MCI from those MCI that will convert to AD eventually. In conclusion, we identified a group of serum proteins with Micro-Western Array platform which may correlate to disease progression of AD and are potential diagnostic serum protein biomarkers.

## 4. Materials and Methods

### 4.1. Recruitment of Participants

Participants of dementia due to AD, their adult children and unrelated healthy control were recruited from the neurological outpatient department of Kaohsiung Municipal Ta-Tung Hospital, located in Southern Taiwan since March 2020. We modified the ACS study [47] for applying in Taiwan. The average age of dementia diagnosed in Taiwan is 79, while their children’s age is around 55 [48]. Participants aged 50–74 years old who have at least one biological parent with probable AD followed up regularly at our outpatient department, were recruited as adult children (AC) group. Participants with depression or already having dementia were excluded. Stable AD patients who had been receiving acetylcholinesterase inhibitors for at least 12 months were recruited as AD group. The diagnosis of AD was based on the National Institute of Neurological and Communicative Disorders and Stroke-Alzheimer’s Disease and Related Disorders Association (NINCDS-ADRDA) criteria [49]. Cognitively normal individuals without dementia as determined by a Clinical Dementia Rating (CDR) [50] score of 0 and an instrument of ascertainment of dementia 8 (AD8) [51] score of <2, and not screened for family history of AD, were recruited in the healthy control (HC) group. The HC individuals recruited in our study were volunteers selected from outpatients at the neurology clinic. The participants and their relatives were informed of the details of study. The Kaohsiung Medical University Hospital Institutional Review Board (KMUHIRB-SV(I)-20190025 and KMUHIRB-G(I)-20210026) approved the study protocol and the participants provided written informed consent prior to their inclusion. The demographic and clinical characteristics of the participants of AD, AC and HC were listed in Table 1. The diagnosis of AD was based on the diagnostic criteria of NINCDS-ADRDA in which psychometrics was being performed to support the diagnosis. The MMSE was also used to support the diagnosis but not for replacement of the diagnosis.

### 4.2. Clinical and Cognitive Assessments

For each recruited participant, a series of neuropsychological assessments, including the AD8, Mini-Mental State Examination (MMSE) [52], CDR and Center for Epidemiological Studies Depression Scale (CESD), were administered to evaluate the clinical, depression status and cognitive function. The neuropsychological assessments were conducted by a senior neuropsychologist and an experienced physician based on information from a knowledgeable collateral source (usually a spouse or adult child).

### 4.3. Micro-Western Array

The profile of interested proteins in the participants’ sera was examined by Micro-Western Array. Sera of participants were lyzed with Micro-Western Array lysis buffer (240 mM Tris-acetate, 1% SDS, 5 mM EDTA and 0.5% glycerol) with protease inhibitor (K272-5, Biovision, Milpitas, CA, USA), phosphatase inhibitor (K275-1EA, Biovision), DTT and 1 mM NA3VO4 as previously described [10]. GeSim Nanoplotter arrayer was used to spot protein lysate, GE multiphor was used for dry electrophoresis, and proteins were transferred onto nitrocellulose membrane [10]. The membrane was blocked with 100% Licor buffer for 1 h and was incubated in primary antibodies diluted in 100% Licor buffer overnight at 4 °C. Next, the membrane was washed three times for 10 min in TBST, and the membrane was incubated for 1 h with Licor fluorescent secondary antibody in 20% Licor buffer and 80% TBS at room temperature. The membrane was washed three times for 10 min in TBST, one time in TBS for 10 min, and air dried. Finally, the membrane was scanned by the Licor Odyssey and was analyzed with Licor Odyssey analysis software (V3.0) [10]. Antibodies for ABCA1, ABCG1, c_Myc and SREBP1 were from abcam (Cambridge, UK); Antibodies for LXRα and LXRβ were from Santa Cruz (Santa Cruz, CA, USA); Antibody for NFκB_p50 was from Millipore (Burlington, MA, USA)

### 4.4. Statistics

Data were presented as the mean ± standard deviation (SD) and proportion. For comparison between AD, ACS and NC groups, the chi-squared test was used for categorical data, and the Kruskal-Wallis test were used for continuous data. The Pearson’s correlation test was used for evaluation of the correlations of plasma proteins and age and MMSE in all groups. Analyses were performed using SPSS 26.0 (SPSS Inc., Chicago, IL, USA). A two-tailed *p*-value of <0.05 was considered to indicate a statistically significant difference. Data were visualized using Prism 7 (Graphpad).

## 5. Conclusions

Using Micro-Western Array, we identified a group of serum proteins which may correlate to the development and the disease progression of AD. We believe that these serum proteins are potential diagnostic serum protein biomarkers for AD.

## Figures and Tables

**Table 1:** Demographic and clinical characteristics of studied participants.

Characteristics	AD (*n* = 30)	AC (*n* = 30)	NC (*n* = 31)	*p* Value			
				Three Groups	AD vs. AC	AD vs. NC	AC vs. NC
Gender, female (%)	80.0	90.0	64.5	<0.001	<0.001	<0.001	<0.001
APOE ε4 positive (%)	46.7	46.7	16.1	<0.001	<0.001	<0.001	<0.001
Age (years)	82.6 ± 6.2	57.5 ± 7.1	74.2 ± 6.0	<0.001	<0.001	<0.01	<0.001
Education (years) ^†^	5.3 ± 5.3	13.7 ± 4.0	10.5 ± 3.8	<0.001	<0.001	<0.01	0.055
MMSE ^‡^	13.6 ± 7.2	28.4 ± 1.6	24.5 ± 3.7	<0.001	<0.001	<0.001	<0.01

Data are shown as the mean ± SD for quantitative variables and *n* (%) for qualitative variables. *p* value for AD, AC and HC group using analysis of chi-square (gender and APOE ε4 positive) or Kruskal-Wallis tests. ^†^ 1 participant missing data (1 AD). ^‡^ 1 participant missing data (1 AC). Abbreviations: AD, Alzheimer’s disease; ACS, adult children of study participants; HC, healthy control; APOE, apolipoprotein E; MMSE, Mini-Mental State Examination

**Table 2:** The correlation of serum proteins with age or Mini-Mental State Examination (MMSE) scores. The Pearson correlation coefficient (r) and corresponding *p*-value are presented for each correlation between the specific protein with age or MMSE.

Proteins	Pearson Correlation Coefficient (r) with Age	*p* Value	Pearson Correlation Coefficient (r) with MMSE	*p* Value
ABCA1	−0.2	0.06	0.014	0.90
ABCG1	0.02	0.81	−0.13	0.23
ApoD	0.04	0.72	−0.003	0.97
ApoE	−0.38 ***	0.0002	0.29 **	0.006
ApoH	−0.15	0.16	−0.15	0.17
c_Myc	−0.39 ***	0.0001	0.37 ***	0.0005
COX2	−0.11	0.31	0.03	0.80
LATS1	−0.38 ***	0.0002	0.27 **	0.01
LXRα	0.04	0.70	0.01	0.93
LXRβ	−0.10	0.34	0.03	0.74
MST1	−0.12	0.24	0.23 *	0.03
MST2	−0.21 *	0.049	0.29 **	0.006
Nanog	−0.16	0.12	0.38 ***	0.0003
NFκB p50	−0.33 **	0.001	0.30 **	0.005
PPARγ	−0.32 **	0.001	0.24 *	0.02
SREBP1	−0.28 **	0.008	0.04	0.67
SREBP2	−0.12	0.26	0.05	0.65
TAZ	−0.16	0.12	0.05	0.66
YAP	−0.06	0.55	0.03	0.81

Pearson’s correlation test was performed to analyze the correlation between the relative expression level of serum proteins with either age or MMSE for all groups of participants. A two-tailed *p*-value of < 0.05 was considered to indicate a statistical significance. Asterisks *, **, *** represented a statistically significant difference of *p* < 0.05, *p* < 0.01, and *p* < 0.005, respectively.

## Data Availability

All data presented in this study are available upon reasonable request.
